# Orthogonal Inverse-Electron-Demand Cycloaddition Reactions Controlled by Frontier Molecular Orbital Interactions

**DOI:** 10.1021/acs.orglett.3c02265

**Published:** 2023-08-17

**Authors:** Dennis Svatunek, Konrad Chojnacki, Titas Deb, Hannah Eckvahl, K. N. Houk, Raphael M. Franzini

**Affiliations:** † Department of Chemistry and Biochemistry, University of California, Los Angeles, Los Angeles, California 90095, United States; ‡ Institute of Applied Synthetic Chemistry, TU Wien, 1060 Vienna, Austria; § Department of Medicinal Chemistry, University of Utah, Salt Lake City, Utah 84112, United States; ∥ Huntsman Cancer Institute, Salt Lake City, Utah 84112, United States

## Abstract

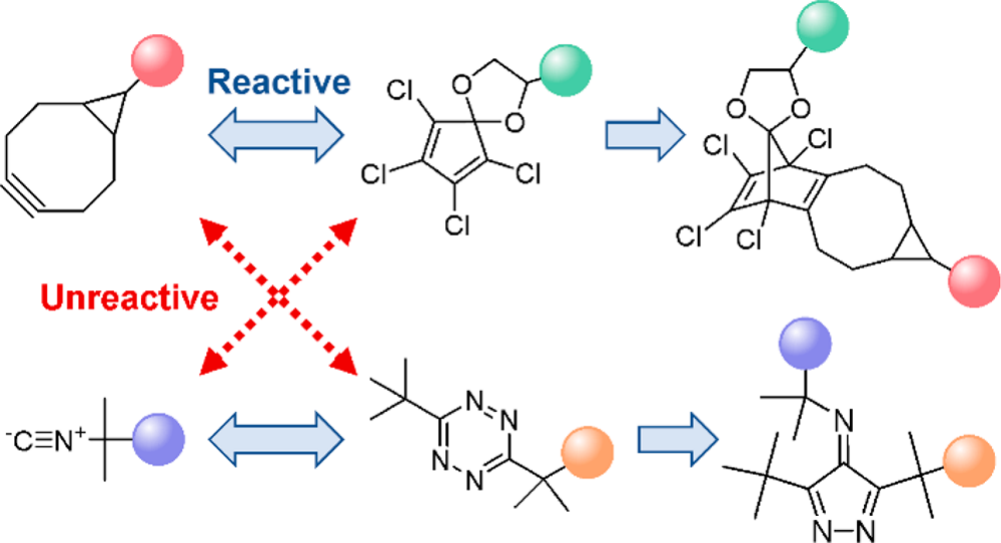

Chemoselective pairs of bioorthogonal reactants enable the simultaneous labeling of several biomolecules. Here, we access orthogonal click reactions by exploiting differences in frontier molecular orbital interaction energies in transition states. We establish that five-membered cyclic dienes are inert to isonitriles but readily react with strained alkynes, while tetrazines with bulky substituents readily react with isonitriles. Strained alkynes show an opposite reactivity pattern. The approach was demonstrated by orthogonally labeling two proteins with different fluorophores.

Bioorthogonal chemistry is ubiquitous at the interface of chemistry and biology.^1^ These reactions provide unprecedented capabilities in the fields of bioconjugation, imaging, biomaterials, and drug delivery. The increasing complexity of biological manipulations often necessitates performing two or more such reactions in parallel, which, in turns, requires orthogonal pairs of bioorthogonal reactants.^2−4^ Several examples of mutually exclusive click reactions have been reported, relying on different strategies to achieve orthogonality (Figure 1). One approach to confer orthogonal reactivity is to use reactions with distinct mechanisms.^5^ For example, the proper choice of reactive groups allows for the performance of [3 + 2]-cycloaddition reactions in parallel with inverse electron-demand Diels–Alder (IEDDA) reactions,^6−8^ and these pericyclic reactions are orthogonal to Staudinger ligation^9^ and aldehyde condensation chemistry.^10^ Another strategy to achieve orthogonality is to purposefully introduce steric clashes to control the reactivity of reactants. As an example, dibenzocyclooctynes undergo a strain-promoted [3 + 2]-cycloaddition with primary azides but not with tertiary azides^11^ or tetrazines.^12^ Similarly, tetrazines with bulky substituents are unreactive to strained alkenes/alkynes but readily react with isonitriles.^13^ Coordinative interactions can also afford selective reactivity.^14^ Further ways of accessing chemoselectivity are required to expand the bioorthogonal chemistry toolbox with a series of reactions that can be used in parallel.

**Figure 1:**
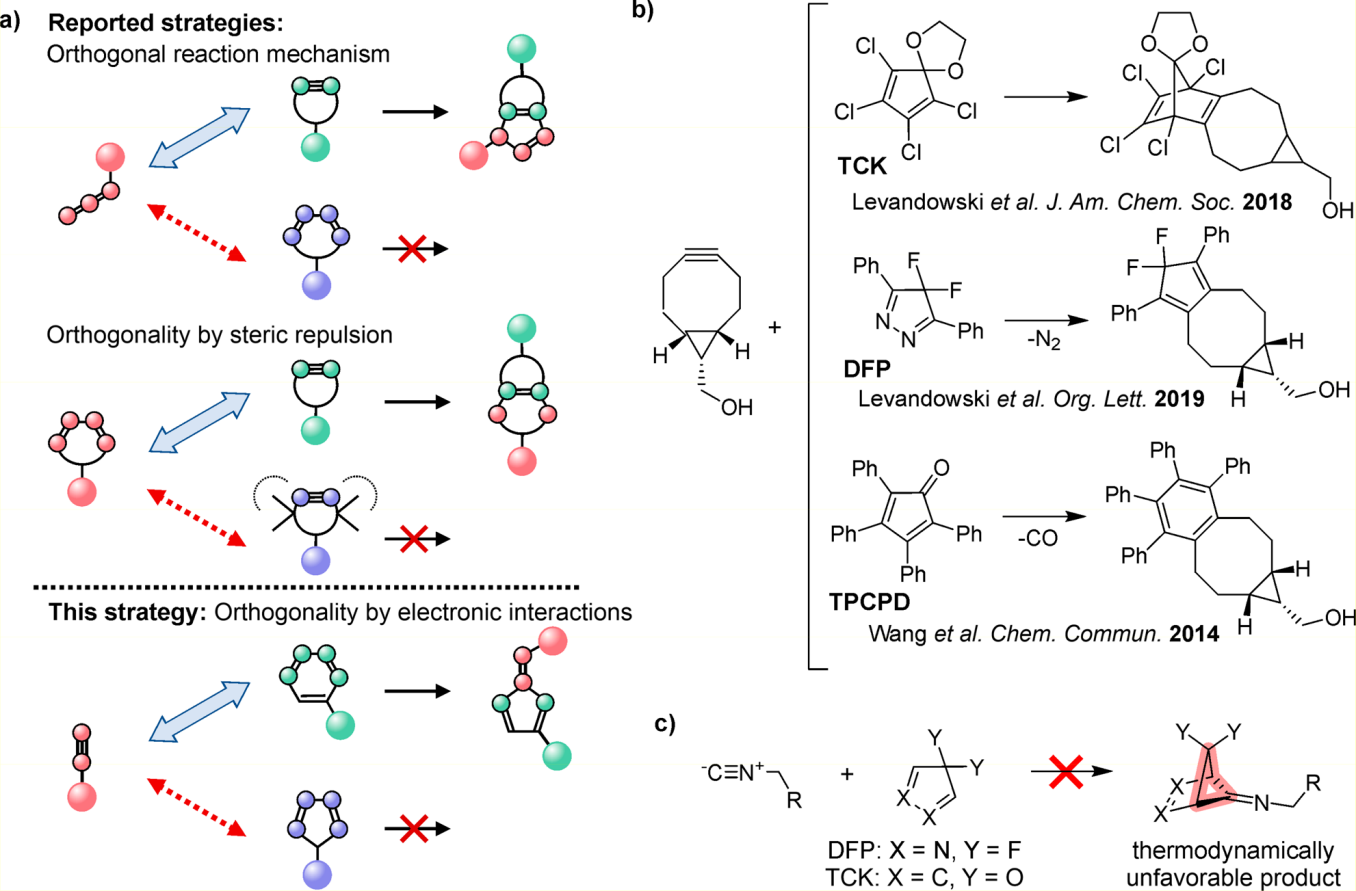
Principle of the outlined approach to achieve mutual orthogonality among bioorthogonal reactions. (a) Examples of strategies used to confer orthogonality to the bioorthogonal reaction. Here, different electronic interactions in transition states are used to achieve chemoselectivity. (b) Reaction of FMCDs with strained alkynes (and alkenes) forms stable adducts. (c) Initial rationale predicted FMDCs to be inert to isonitriles because the [4 + 1]-cycloaddition reaction would generate a thermodynamically unfavorable product because of the formation of a highly strained four-membered ring.

Here, we exploit differences in the interaction energy during transition states to confer orthogonality to bioorthogonal IEDDA reactions between electron-deficient dienes and dienophiles (Figure 1a). The most widely used dienes in IEDDA reactions are 1,2,4,5-tetrazines that undergo [4 + 2]-cycloadditions with strained alkenes and alkynes forming pyridazines^15^ but also react with isonitriles, generating 4-aminopyrazoles.^16−19^ In addition, several five-membered cyclic dienes (FMCDs) have been reported in the context of IEDDA reactions (Figure 1b). Tetrachlorocyclopentadienone ethylene ketal (TCK) reacts with strained alkynes and alkenes to form stable adducts,^20^ and 4,4-difluoro-3,5-diphenyl-4*H*-pyrazole (DFP) reacts with strained alkynes forming a cyclopentadiene conjugate.^20,21^ Relatedly, cyclopentadione (TPCPD)^22^ and thiophene dioxide^23^ react with strained alkynes to generate a benzene ring under the release of CO and SO_2_, respectively. Recently, oxidized tellurophene was used for IEDDA-based conjugation.^24^ Cognizant of the IEDDA rection of FMCDs with strained triple bonds, we envisioned that such dienes would be unreactive to isonitrile dienophiles, which would open possibilities for developing orthogonal bioorthogonal reactions. The initial rationale was that isonitriles contribute only one carbon atom to the new ring, which in the case of TCK and DFP should form unfavorably strained four-membered rings (Figure 1c). While experimental studies confirmed the orthogonality between isonitriles and FMCDs, detailed computational analyses revealed that inefficient frontier molecular orbital interactions are a main reason for this outcome.

We tested the concept of chemoselective IEDDA reactions based on different ring sizes of dienes using high-performance liquid chromatography (HPLC) analysis. TCK and DFP were synthesized as reported^21,25^ and exposed to either a fluorophore-labeled bicyclononyne (5-FAM-BCN; structures are shown in Figure S1 in the Supporting Information) or isonitrile (BDPY-FL-NC), and the reaction mixture was analyzed by HPLC. The reaction of TCK with 5-FAM-BCN [*c*(BCN) = 2.5 mM, *c*(TCK) = 10 mM, dimethyl sulfoxide (DMSO), *t* = 24 h, and *T* = 37 °C] resulted in the disappearance of starting material and emergence of a fluorophore-containing species in agreement with literature reports (Figure 2).^21,25^ In contrast, the starting materials remained intact when TCK was mixed with BDPY-FL-NC under the same conditions (Figure 2). Similarly, DFP readily reacted with BCN but not with DFP (Figure S2 of the Supporting Information). No reaction was observed between TCK and phenylethylisonitrile in 8:2 DMSO-*d*_6_/D_2_O within 24 h (Figure S4 of the Supporting Information). These experiments confirm that FMCDs selectively react with strained alkynes while being orthogonal to isonitrile dienophiles.

**Figure 2:**
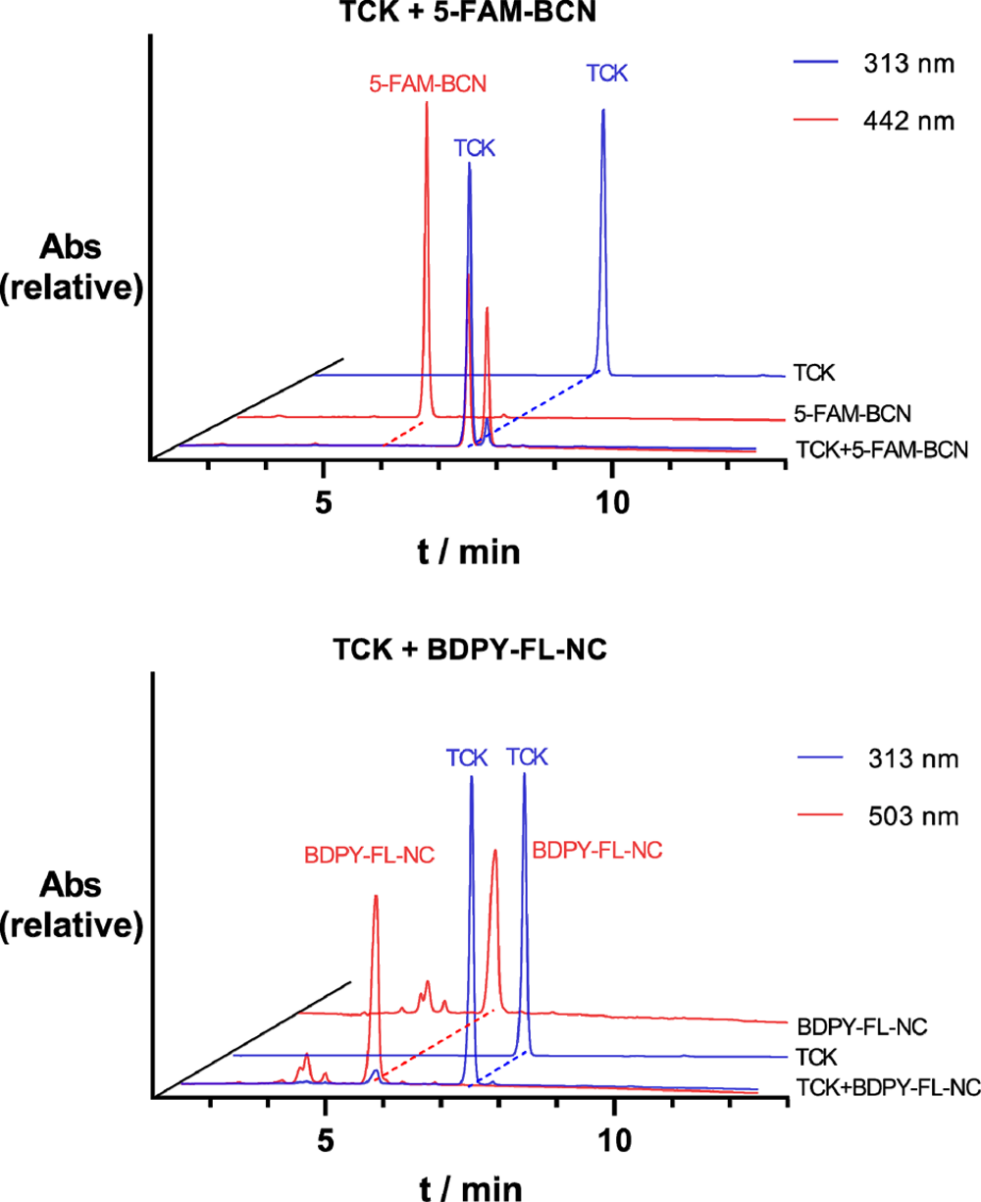
Test of the reaction between fluorophore-labeled bicyclononyne (5-FAM-BCN) or isonitrile (BDPY-FL-NC) and TCK [structures are shown in Figure S1 of the Supporting Information; conditions: *c*(BCN or NC) = 2.5 mM, *c*(TCK) = 10 mM, DMSO, *t* = 24 h, and *T* = 37 °C].

To gain an understanding of the orthogonal reactivity, a computational study was conducted. M06-2X, a density functional known to perform well for cycloadditions,^26−28^ was used together with the 6-311+G(d,p) basis set and the SMD water model. Calculations were executed using Gaussian 16, Revision A.03,^29^ and entropies were corrected using a quasi-harmonic approximation in GoodVibes.^30^

First, the potential energy surface of the reaction between isonitriles and cycloocytnes with TCK was explored in the gas phase. Methyl isocyanide (MeNC) was used as a model compound, and a truncated BCN* was used as the cyclooctyne. Both reactions were found to proceed through a concerted transition state with free energy barriers of 14.5 and 35.4 kcal/mol for BCN* and MeNC, respectively (Figure 3a). This result is consistent with experimental observations, where BCN reacts rapidly with cyclopentadiene, while isonitrile is unreactive. The difference in barrier height can be attributed to the energy required to distort TCK into the respective transition state geometries. A distortion/interaction analysis^31,32^ reveals that, in case of the isonitrile cycloaddition, the distortion energy (Δ*E*_dist_) for the diene is 30.3 kcal/mol, while for BCN*, it is only 9.4 kcal/mol in agreement with the initial hypothesis that ring strain would disfavor the reaction.

**Figure 3:**
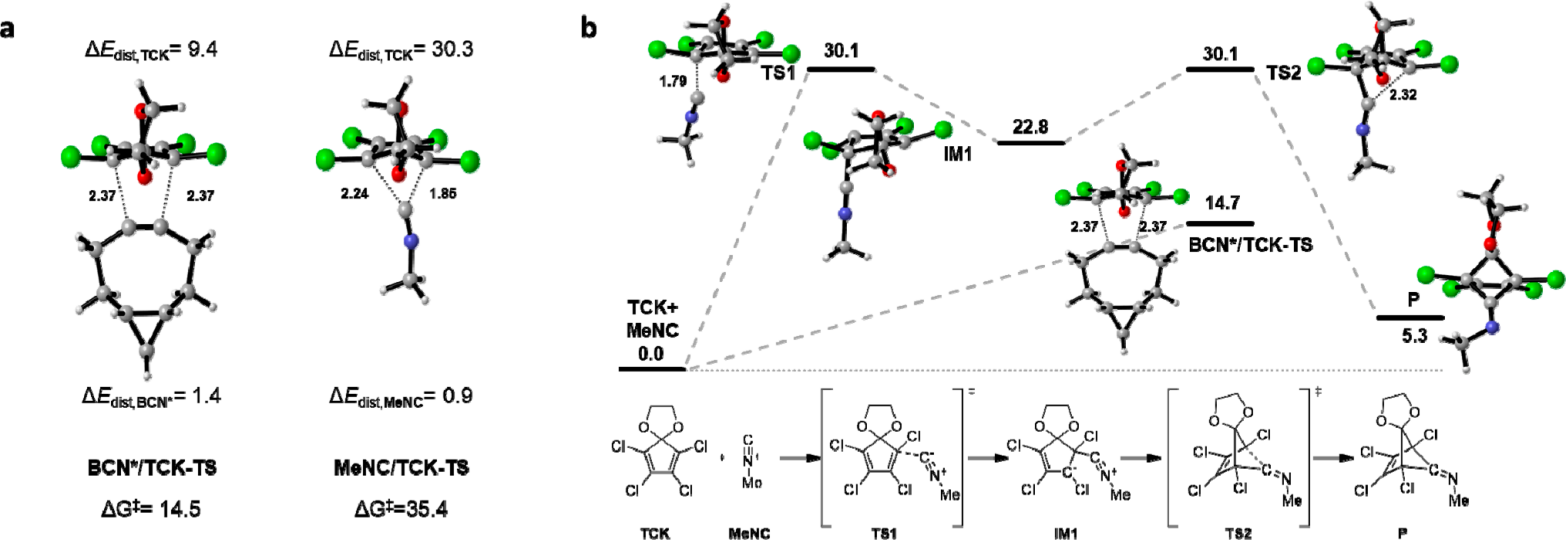
(a) Gas-phase transition states for BCN* and MeNC with TCK. Distortion energies reveal that the high barrier of MeNC + TCK is caused by the substantial energy penalty of distorting TCK into the transition state geometry. (b) Calculated reaction pathway of TCK with methyl isocyanide in water. The barrier for the initial nucleophilic attack in TS1 is prohibitively high for the reaction to proceed at room temperature. Additionally, the reaction is endergonic. For comparison, the transition state energy of the [4 + 2]-cycloaddition reaction of TCK with bicyclononyne (BCN*) with a barrier of 14.7 kcal/mol is shown, demonstrating that this reaction can readily occur under ambient conditions. All energies are in kcal/mol.

However, MeNC additionally shows a considerably later transition state in the reaction with TCK than with BCN*. Typically, such late transition states are accompanied by a higher distortion energy, which prevents us from definitively attributing ring strain to the cause for the higher barrier. The late transition state is, however, unquestionably caused by a weaker interaction energy, necessitating a close approach for sufficient orbital overlap and interaction to occur. Therefore, we conclude that the elevated barrier results from the weak dienophile–diene interaction, leading to a late and substantially distorted transition state. An analysis of the frontier molecular orbital (FMO) interactions reveals that BCN* has a considerably higher highest occupied molecular orbital (HOMO) with calculated values of −8.26 eV compared to −10.65 eV for MeNC, which explains the weaker interaction of isonitrile with FMCDs (Table S1 of the Supporting Information). No significant normal electron-demand interaction was observed.

In aqueous solution, the MeNC/TCK cycloaddition proceeds through a two-step process, with an initial nucleophilic attack and a subsequent cyclization (Figure 3b). The initial addition has a barrier of 30.1 kcal/mol, resulting in a high-energy zwitterionic intermediate with an energy of 22.8 kcal/mol relative to starting materials. Although this stepwise process is usually disfavored over the concerted reaction, in this case, it allows the system to avoid the highly strained concerted transition state that is a result of the weak interaction between the dienophile and diene. Unlike in the gas phase, solvent interactions sufficiently stabilize the zwitterionic intermediate to favor this pathway over a concerted reaction. A second barrier of 7.3 kcal/mol leads to a thermodynamically unfavorable product with a free energy of 5.3 kcal/mol relative to the starting material. In contrast, the influence of a solvent system on the BCN*/TCK system is negligible. The high barriers and thermodynamically unstable product, caused by the high strain in the four-membered ring structure of the product, prevent the reaction between isonitriles and TCK. With regard to DFP, a thermodynamically stable product is formed after elimination of dinitrogen in a retro-Diels–Alder reaction. However, once again, high barriers prevent this reaction from occurring under physiological conditions (Figure S3 of the Supporting Information).

Interestingly, despite the weak interaction between MeNC and TCK, leading to no reaction, MeNC rapidly reacts with sterically demanding 1,2,4,5-tetrazine dienes, such as 3,6-bis-*tert*-butyl-1,2,4,5-tetrazine, as previously evidenced.^13^ However, this particular tetrazine has a higher unoccupied orbital energy (−0.4 eV) compared to TCK (−1.20 eV), leading to even weaker orbital interactions. This reactivity, not governed by the frontier molecular orbital interaction, can be attributed to a reduction in Pauli repulsion in the case of tetrazine compared to TCK, leading to a stronger interaction energy (refer to the Supporting Information for a comprehensive analysis). The rate-enhancing effect of lowered Pauli repulsion in 1,2,4,5-tetrazines has been previously described by us and others.^33,34^

Therefore, our computational study suggests that MeNC is unreactive toward TCK because of weak orbital interactions, whereas BCN* reacts rapidly as a result of its significantly higher HOMO. Conversely, MeNC reacts favorably with tetrazines, owing to the reduced Pauli repulsion related to the nucleophilic interaction on the tetrazine carbons, while BCN* is unable to react with sterically demanding tetrazines as a result of steric hindrance.

We performed protein-labeling experiments to demonstrate the orthogonality of the IEDDA reaction of isonitriles and strained alkynes with tetrazines and FMCDs (Figure 4). Two proteins were modified with different dienes: bovine serum albumin (BSA) was modified with a tetrazine molecule containing two tertiary alkyl substituents (BSA-Tz), and TCK was conjugated to ovalbumin. Bis-*tert*-butyl-substituted tetrazines are unreactive to strained alkynes and alkenes because of steric clash but readily react with isonitriles.^13^ The preparation of BSA-Tz was reported,^13^ and synthesis of the carboxylic acid derivative of TCK and its conjugation to lysine residues on ovalbumin were performed according to published protocols.^20^ Modified proteins were incubated with fluorophore-labeled bicyclononyne (BCN-SiR) and isonitrile (BDPY-FL-NC), and the labeling reaction was analyzed by sodium dodecyl sulfate (SDS) protein gel analysis. One-pot exposure of BSA-Tz to SiR-BCN and BDPY-FL-NC yielded a distinctly green fluorescent band indicative of selective reaction with the isonitrile probe. Conversely, ovalbumin exclusively reacted with the strained alkyne probe, as indicated by the red fluorescence staining. A mixture of both proteins provided orthogonally labeled BSA and an ovalbumin band. These experiments confirm the orthogonal bioconjugation reactions based on different ring sizes in IEDDA reactions.

**Figure 4:**
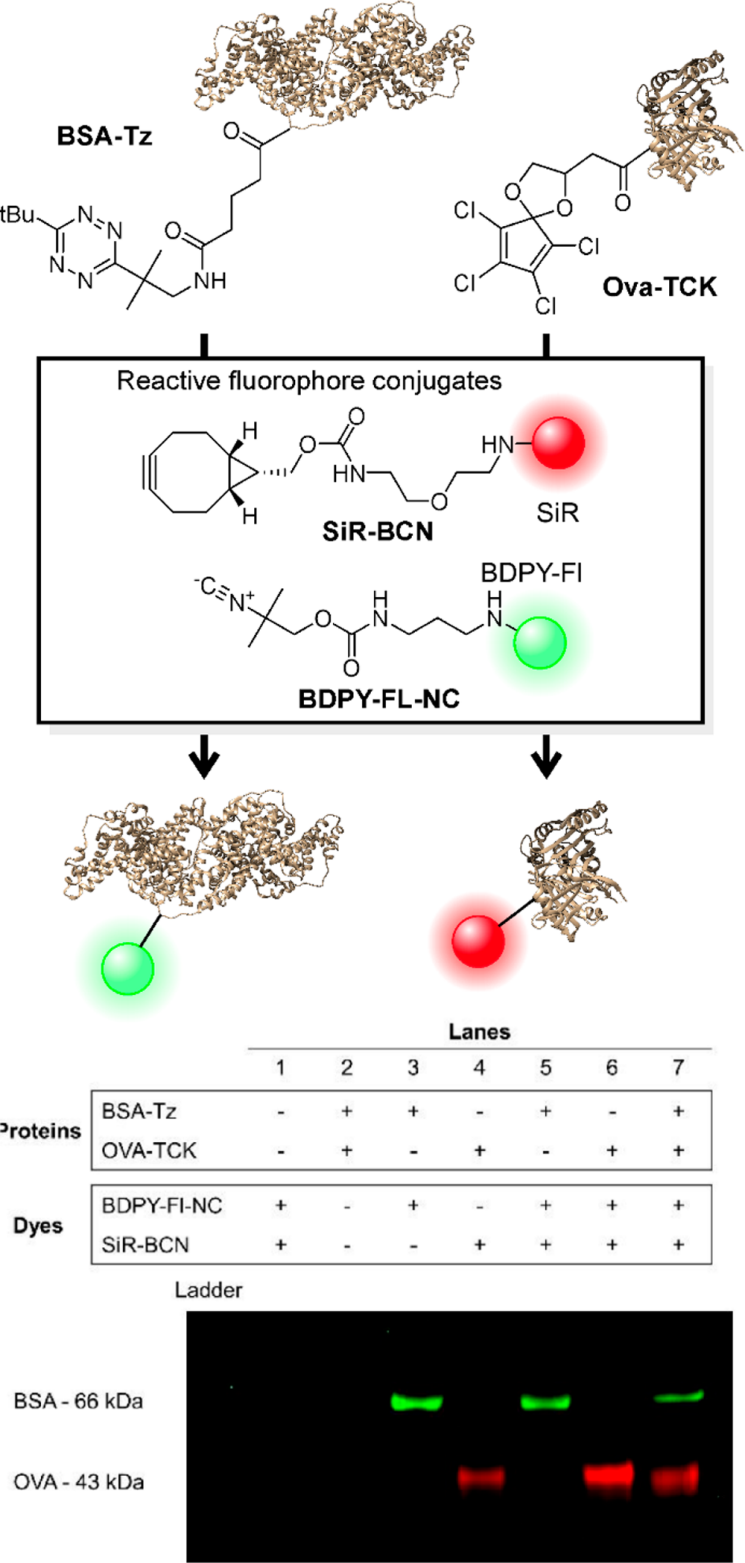
Orthogonal labeling of proteins based on mutually exclusive reactions of isocyanides with sterically bulky tetrazines and BCN with TCK. (a) Reaction scheme. (b) Gel electrophoresis analysis of mutually exclusive protein labeling.

In conclusion, we have introduced a novel bioorthogonal reactant pair that exhibits orthogonal reactivity based on the IEDDA mechanism. Pairs of sterically encumbered tetrazines and isonitriles have orthogonal reactivity to cyclooctyne dienophiles and DFP or TCK dienes, as confirmed in protein-labeling experiments. Importantly, our computational analysis reveals that the reaction of isonitriles with FMCDs is energetically unfavorable as a result of the substantial HOMO/lowest unoccupied molecular orbital (LUMO) gap and the strained four-membered ring system in the resulting product.

## Data Availability

The data underlying this study are available in the published article and its Supporting Information.

## Supporting Information Available

Cartesian coordinates of all structures as machine-readable XYZ files (ZIP)
Experimental protocols and supplemental data (PDF)
