# Supplementary material for: Orthogonal Inverse-Electron-Demand Cycloaddition Reactions Controlled by Frontier Molecular Orbital Interactions
Source: Org Lett. 2023 Aug 17;25(34):6340–5. doi: 10.1021/acs.orglett.3c02265 (PMC10476241; doi:10.1021/acs.orglett.3c02265)
Supplement: Supplementary file 2 — ol3c02265_si_002.pdf [file ol3c02265_si_002.pdf]

Supporting Information

Orthogonal Inverse-electron Demand Cycloaddition Reactions Controlled by Frontier Molecular Orbital Interactions

Dennis Svatunek, Konrad Chojnacki, Hannah Eckvahl, Titas Deb, K. N. Houk, Raphael M. Franzini*

D. Svatunek - Department of Chemistry and Biochemistry, University of California, Los Angeles, California 90095, USA; Institute of Applied Synthetic Chemistry, TU Wien, 1060, Vienna, Austria

K. Chojnacki – Department of Medicinal Chemistry, University of Utah, Salt Lake City, Utah 84112, USA

H. Eckvahl – Department of Chemistry and Biochemistry, University of California, Los Angeles, California 90095, USA

T. Deb – Department of Medicinal Chemistry, University of Utah, Salt Lake City, Utah 84112, USA

K. N. Houk - Department of Chemistry and Biochemistry, University of California, Los Angeles, California 90095, USA

R. M. Franzini – Department of Medicinal Chemistry, University of Utah, Salt Lake City, Utah 84112, USA

Table of Contents

General Information 2
Synthesis of 4,4-difluoro-3,5-diphenyl-4H-pyrazole (DFP) 2
Synthesis of Tetrachlorocyclopentadienone ethylene ketal (TCK) 2
HPLC Analyses 2
DFT Calculation 4
Protein Labeling Experiments 7
References 8

Figures and Tables

Fig. S1. Structures of IEDDA reactants used in this study 3
Fig. S2. HPLC analysis of the reaction between 5-FAM-BCN or BDPY-FL-NC and DFP 3
Fig. S3. Potential energy profile of the reaction between DFP and MeNC in SMD water 4
Fig. S4. Prolonged study about the reaction of TCK and isonitriles 5
Table S1. M06-2X/6-311+G(d,p) calculated energies for all investigated reactions 6
Table S2. Energy Decomposition Analysis for the reaction of MeNC with TCK and tBu_2Tz 7

General information

All solvents were reagent grade or HPLC grade. Unless otherwise noted, all materials were obtained from commercial suppliers and used without further purification. Tetrachlorocyclopentadienone ethylene ketal (TCK) and 4,4-difluoro-3,5-diphenyl-4*H*-pyrazole (DFP) were synthesized according to the literature procedures and their spectroscopic characterization were consistent with reported data.[1, 2] Fluorophore labeled isonitrile (BDPY-FL-NC) was previously synthesized in our group.[3] Fluorophore labeled bicyclononyne (5-FAM-BCN) was purchased from a commercial source (Conju-Probe, LLC; SKU: CP-4020).

HPLC analyses were performed on an UltiMate3000 HPLC (Thermo Fisher Scientific) equipped with UV-Vis diode array detector, using C18 reversed-phase column (Luna 5u C18, 150 x 2.00 mm, flow 1 ml/min) and elution with gradient mixture of water (0.1% trifluoroacetic acid) / acetonitrile.

Synthesis of 4,4-difluoro-3,5-diphenyl-4*H*-pyrazole (DFP)

DFP was synthesized according to the previously reported procedure.[1] In the oven-dried flask solution of 3,5-diphenylpyrazole (210 mg, 0.94 mmol, 1.0 eq.) and 1-chloromethyl-4-fluoro-1,4-diazoniabicyclo[2.2.2]octane bis(tetrafluoroborate) (770 mg, 2.06 mmol, 2.19 eq.) along with molecular sieves 3Å in acetonitrile (3 mL) was heated at 90°C under Ar for 1 h. The reaction mixture was diluted with ethyl acetate (5 mL), filtered, and solid was washed with ethyl acetate (3 x 5 mL). Filtrate was concentrated under reduced pressure and the product was purified by silica chromatography (0-10% v/v ethyl acetate in hexanes). 158 mg (65%) of DFP as light-yellow solid was obtained. **^{1}H NMR** (400 MHz, $CDCl_3$, δ): 8.07-8.21 (m, 4H), 7.49-7.68 (m, 6H).

Synthesis of Tetrachlorocyclopentadienone ethylene ketal (TCK)

TCK was synthesized according to the previously reported procedure.[2] To a solution of hexachlorocyclopentadiene (412 mg, 1.5 mmol, 1.0 eq.) in ethylene glycol (1 mL) at 0°C was added dropwise solution of KOH (226 mg, 3.83 mmol, 2.56 eq.) in ethylene glycol (2 mL). The reaction mixture was allowed to warm up to RT and stirred overnight. The reaction mixture was diluted with water (10 mL) and extracted with diethyl ether (3 x 10 mL). Combined organic layers were dried over $MgSO_4$, filtered, and concentrated under reduced pressure. The product was purified by silica chromatography (0-2% v/v ethyl acetate in hexanes). 102 mg (26%) of TCK as white solid was obtained. **^{1}H NMR** (400 MHz, $CDCl_3$, δ): 4.32 (s).

HPLC analyses

Mixtures of 5-FAM-BCN or BDPY-FL-NC (2.5 mM) and TCK or DFP (10 mM) in DMSO were incubated at 37°C for 24 h. Sample of each mixture (4 µl) was diluted with acetonitrile (16 µl) and analyzed by HPLC. Detection at 290 nm (DFP), 313 nm (TCK), 442 nm (5-FAM-BCN) and 503 nm (BDPY-FL-NC).

Specific HPLC method for each mixture with percentage of acetonitrile and retention time are as follows:

DFP/5-FAM-BCN: HPLC (C18, 0.1% TFA in water/acetonitrile = 75/25 (0-1 min), 75/25 - 65/35 (1-15 min), 65/35 – 25/75 (15-20 min), 25/75 – 0/100 (20-22 min), 0/100 (22-23 min), flow rate = 1.0 mL/min, l = 290 nm (DFP) and 442 nm (5-FAM-BCN)) tR = 12.4 min (DFP), 14.2 min (5-FAM-BCN)

DFP/BDPY-FL-NC: HPLC (C18, 0.1% TFA in water/acetonitrile = 75/25 (0-1 min), 75/25 - 25/75 (1-8 min), 25/75 – 0/100 (8-10 min), 0/100 (10-11 min), flow rate = 1.0 mL/min, l = 290 nm (DFP) and 503 nm (BDPY-FL-NC)) tR = 5.3 min (BDPY-FL-NC), 5.5 min (DFP).

TCK/BDPY-FL-NC: HPLC (C18, 0.1% TFA in water/acetonitrile = 75/25 (0-1 min), 75/25 - 25/75 (1-8 min), 25/75 – 0/100 (8-10 min), 0/100 (10-11 min), flow rate = 1.0 mL/min, l = 313 nm (TCK), 503 nm (BDPY-FL-NC)) tR = 5.3 min (BDPY-FL-NC), 7.0 min (TCK).

TCK/5-FAM-BCN: HPLC (C18, 0.1% TFA in water/acetonitrile = 75/25 (0-1 min), 75/25 - 25/75 (1-8 min), 25/75 – 0/100 (8-10 min), 0/100 (10-11 min), flow rate = 1.0 mL/min, **l** = 313 nm (TCK), 442 nm (5-FAM-BCN)) tR = 5.3 min (5-FAM-BCN), 7.0 min (TCK).

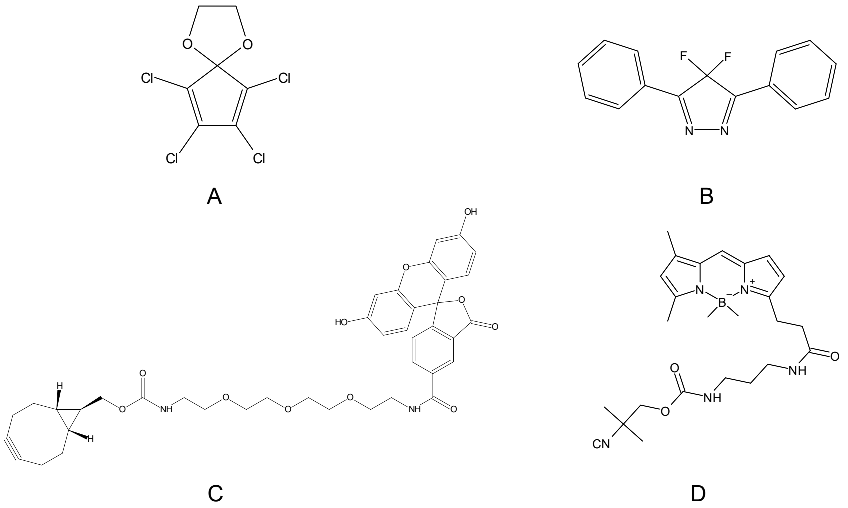

Fig. S1. Structures of IEDDA reactants used in this study: TCK (A), DFP (B), 5-FAM-BCN (C), BDPY-FL-NC (D).

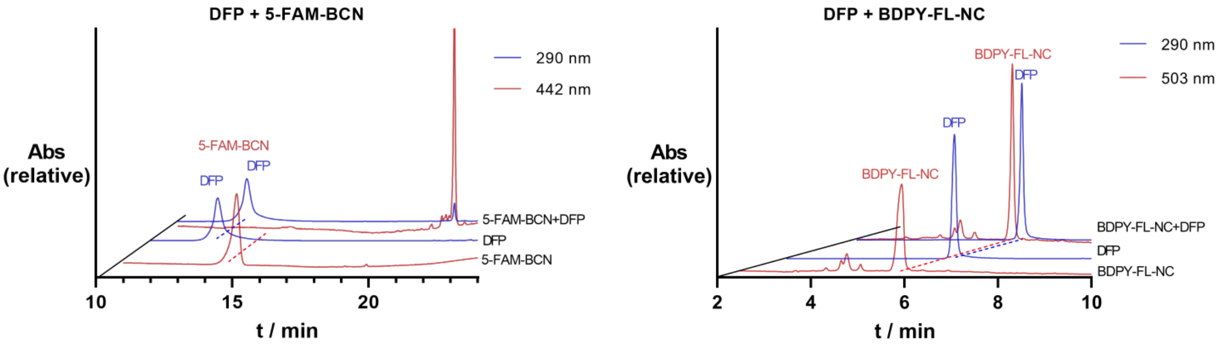

Fig. S2. HPLC analysis of the reaction between fluorophore-labeled bicyclononyne (5-FAM-BCN) or isonitrile (BDPY-FL-NC) and DFP. Conditions: c(BCN or NC) = 2.5 mM, c(DFP) = 10 mM, DMSO, t = 24 h, T = 37°C).

DFT calculations

Figure S3 shows the potential energy profile for the reaction of **DFP** with **MeNC** in water solvation.

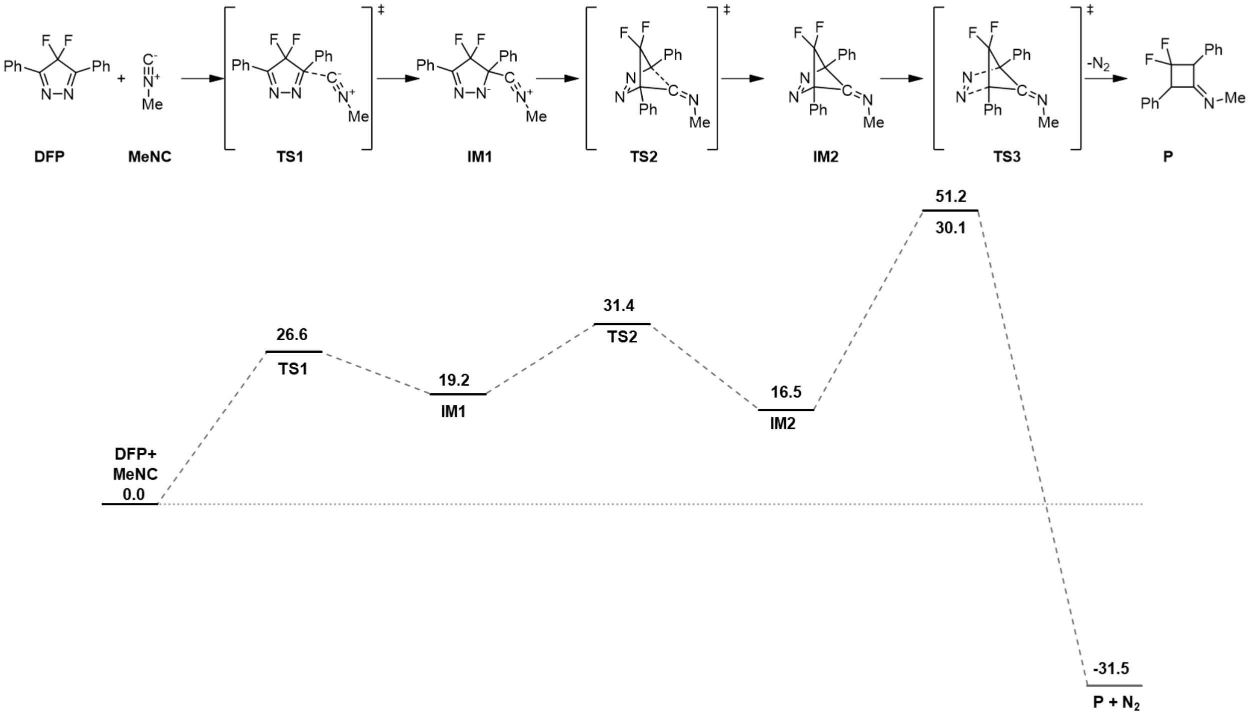

Fig S3. Potential energy profile of the reaction between **DFP** and **MeNC** in SMD water. All energies in kcal/mol.

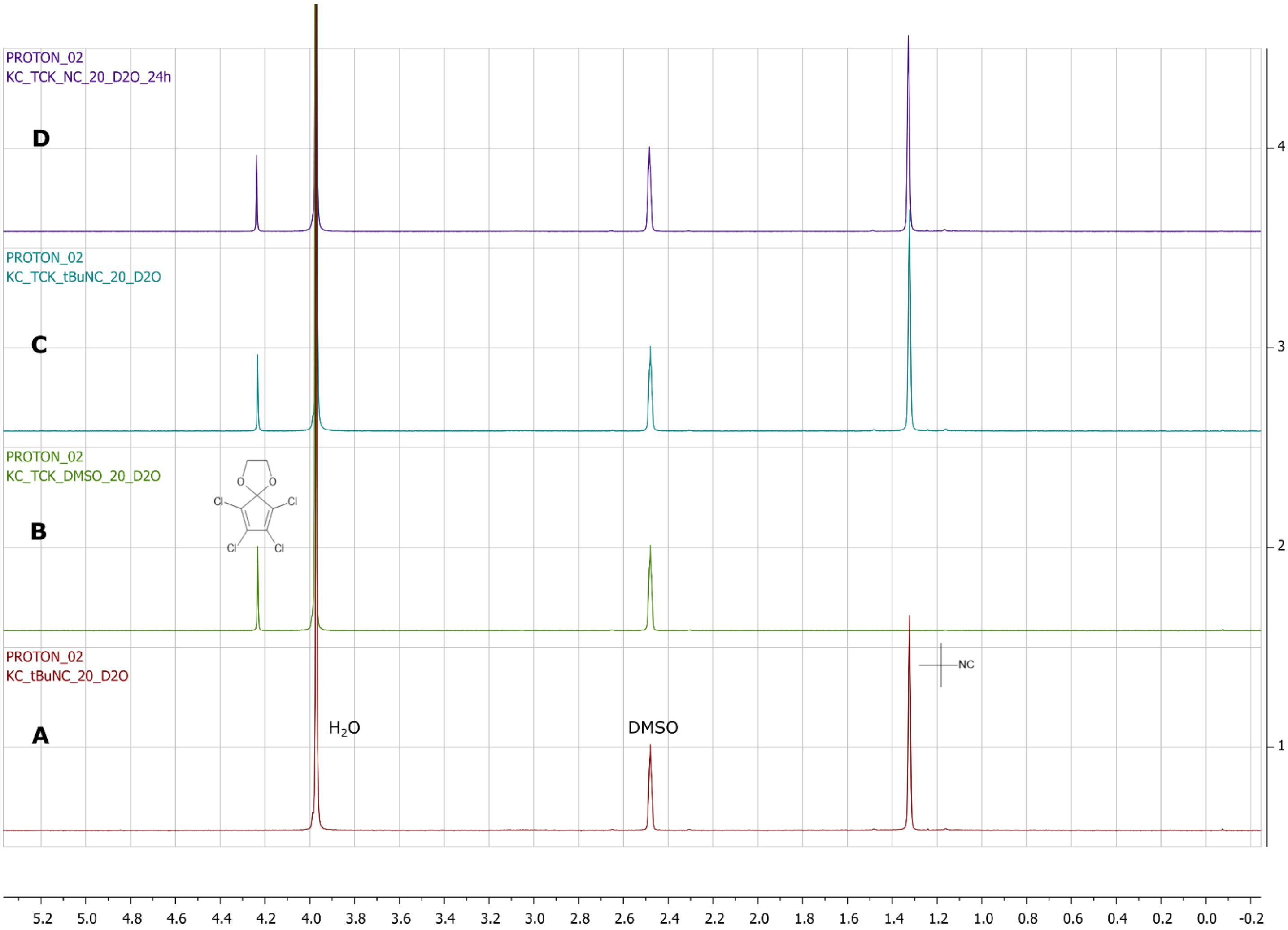

Fig S4. Prolonged study about the reaction of TCK and isonitriles. ^{1}H NMR monitoring of the non-reaction between TCK and ^{t}BuNC in DMSO-d_6 / D_2O 8:2 (*v*/*v*): (**A**) 12 mM ^{t}BuNC, (**B**) 3 mM TCK, (**C**) 3mM TCK + 12 mM ^{t}BuNC after 5 min., (**D**) 3mM TCK + 12 mM ^{t}BuNC after 24 h.

Table S1. M06-2X/6-311+G(d,p) calculated energies for all investigated reactions in gas phase and SMD water and the number of imaginary frequencies (n_{imag}).

Table S1 lists energies for all calculated structures. Local minima structures were identified by 0 imaginary frequencies, while transition states were identified by exactly one imaginary frequency. **MeNC-TCK TS1** has a second negative frequency of -9 cm^{-1} which could not be eliminated. It is associated with a rotation of the isonitrile methyl group and is due to very limited interaction with other groups and the linear nature of the isonitrile is in a very flat region of the potential energy surface. As this imaginary frequency is very close to 0 it was ignored.

Structure	ΔE (hartree)	ZPE (hartree)	ΔH_{298} (hartree)	ΔG_{298} (hartree)	n_{imag}
gas phase					
**BCN**	-350.010971	0.187109	-349.815018	-349.855676	0
**MeNC**	-132.697312	0.045709	-132.646895	-132.675707	0
**TCK**	-2260.271988	0.101517	-2260.158640	-2260.208696	0
**BCN*-TCK TS**	-2610.283261	0.289441	-2609.972010	-2610.041292	1
**MeNC-TCK TS**	-2392.932535	0.148312	-2392.766711	-2392.827982	2
SMD water					
**BCN**	-350.015622	0.186443	-349.820304	-349.860999	0
**MeNC**	-132.698898	0.045800	-132.648425	-132.677162	0
**TCK**	-2260.277523	0.101637	-2260.164041	-2260.214101	0
**DFP**	-886.656493	0.218240	-886.422581	-886.478759	0
**N_2**	-109.522715	0.005761	-109.513650	-109.535377	0
**BCN*-TCK TS**	-2610.293361	0.289148	-2609.982444	-2610.051641	1
**BCN*-DFP TS1**	-1236.670401	0.404424	-1236.241971	-1236.314014	1
**BCN*-DFP IM1**	-1236.746256	0.408896	-1236.314374	-1236.384299	0
**BCN*-DFP TS2**	-1236.738033	0.406424	-1236.308326	-1236.379011	1
**BCN*-DFP P**	-1127.293947	0.397444	-1126.874881	-1126.942492	0
**MeNC-TCK TS1**	-2392.948643	0.148266	-2392.783644	-2392.843354	1
**MeNC-TCK IM1**	-2392.960931	0.149640	-2392.793649	-2392.854916	0
**MeNC-TCK TS2**	-2392.949270	0.149205	-2392.783118	-2392.843311	1
**MeNC-TCK P**	-2392.991366	0.151415	-2392.823393	-2392.882799	0
**MeNC-DFP TS1**	-1019.332742	0.263824	-1019.049070	-1019.113601	1
**MeNC- DFP IM1**	-1019.345510	0.264816	-1019.060888	-1019.125257	0
**MeNC- DFP TS2**	-1019.325945	0.264312	-1019.042374	-1019.105950	1
**MeNC- DFP IM2**	-1019.352882	0.266813	-1019.067327	-1019.129613	0
**MeNC- DFP TS3**	-1019.292362	0.262215	-1019.010686	-1019.074304	1
**MeNC- DFP P**	-909.883772	0.255728	-909.610287	-909.670800	0

An Energy Decomposition Analysis (EDA)[4] was conducted to explore why **MeNC** reacts with tetrazines but not with **TCK**. We utilized 3,6-Di-tBu-1,2,5,6-tetrazine (**tBu_2Tz**) as the tetrazine model. The analysis was carried out using the ADF 2022.101 package,[5-7] employing the M06-2X functional with a TZP basis set, no frozen core, and a numerical quality of VeryGood. This study was conducted at a consistent geometry

where the shorter forming bond length was frozen at 1.96 Å. The results of the energy decomposition analysis are provided in Table S2.

As anticipated, based on the Frontier Molecular Orbital consideration where the tetrazine presents the higher unoccupied orbital, the orbital interaction (ΔE_{oi}) is weaker for **tBu_2Tz**. Electrostatic interactions (ΔV_{elstat}) also favor the **TCK** system. However, the Pauli repulsion (ΔE_{Pauli}) is significantly less unfavorable in the tetrazine system, resulting in an overall much stronger interaction energy (ΔE_{int}).

Table S2. Energy Decomposition Analysis for the reaction of **MeNC** with **TCK** and **tBu_2Tz**.

Structure	ΔE_{Pauli} (kcal/mol)	ΔV_{elstat} (kcal/mol)	ΔE_{oi} (kcal/mol)	ΔE_{int} (kcal/mol)
**MeNC + TCK**	153.58	-81.57	-72.72	-0.71
**MeNC + tBu_2Tz**	127.09	-75.08	-65.21	-13.2

Protein labeling experiments

Ovalbumin from chicken egg (**OVA**) was purchased commercially (Research Products International) and was used without further purification. Fluorophore-labeled bycyclononyne (**SiR-BCN**) was purchased commercially (Spirochrome). 2,5-Dioxopyrrolidin-1-yl 2-(6,7,8,9-tetrachloro-1,4-dioxaspiro[4.4]nona-6,8-dien-2-yl) acetate (**TCK-NHS**) was synthesized according to the literature procedure.[1] The conjugate **BSA-Tz** had previously been synthesized in our group.[3] The protein conjugate (**OVA-TCK**) was synthesized in reaction of TCK-NHS with OVA according to previously reported procedure.[3] Mass analysis showed the labeling efficiency to be a distribution of 9-13 molecules of TCK (302.9 Da) per one molecule of 123 ovalbumin (42.7 kDa), with a maximum at 11. MS (ESI-TOF) m/z: 45539.2, 45837.6, 46142.4, 46384.8, 46688.0.

Protein conjugates, OVA-TCK and BSA-Tz (80 µL of a 2 mg/mL solution in PBS, pH 7.4), were treated with the fluorophore labeled dienophile conjugates, SiR-BCN and BDPY-FL-NC (20 µL of 1 mM solution in DMSO), individually with the corresponding dye or in the presence of both dyes. For dual labeling, the protein conjugates were combined in a 1:1 ratio and labeled with both fluorophores. Reactions were carried out at 37°C for 3 h, followed by removal of small molecules by centrifugation through Amicon Ultra filters with a 10kDa MWCO. Proteins were collected and analyzed by SDS-PAGE. In-gel fluorescence labeling was imaged with the ChemiDoc MP imaging system (Bio-Rad) using Alexa 488 (green) and Alexa 680 (red) filters, and the images processed through the Image Lab software. Quantification of the band intensities was performed using the ImageJ software (https://imagej.nih.gov/ij/). Protein transfer from the gel onto a nitrocellulose membrane was performed using the iBlot 2 Dry Blotting System (ThermoFisher Scientific). The membrane was stained using Ponceau stain for 2 min, followed by washing with deionized water. The image was acquired using the ChemiDoc MP imaging system (Bio-Rad) to assess protein loading.

References

1. Levandowski, B. J.; Abularrage, N. S.; Houk, K. N.; Raines, R. T., Hyperconjugative Antiaromaticity Activates 4H-Pyrazoles as Inverse-Electron-Demand Diels-Alder Dienes. *Org Lett* **2019,** *21* (20), 8492-8495.
2. Levandowski, B. J.; Gamache, R. F.; Murphy, J. M.; Houk, K. N., Readily Accessible Ambiphilic Cyclopentadienes for Bioorthogonal Labeling. *J Am Chem Soc* **2018,** *140* (20), 6426-6431.
3. Tu, J.; Svatunek, D.; Parvez, S.; Liu, A. C.; Levandowski, B. J.; Eckvahl, H. J.; Peterson, R. T.; Houk, K. N.; Franzini, R. M., Stable, Reactive, and Orthogonal Tetrazines: Dispersion Forces Promote the Cycloaddition with Isonitriles. *Angew Chem Int Ed Engl* **2019,** *58* (27), 9043-9048.
4. Hamlin, Trevor A., Vermeeren, Pascal, Guerra, Célia Fonseca and Bickelhaupt, F. Matthias. "8 Energy decomposition analysis in the context of quantitative molecular orbital theory". Complementary Bonding Analysis, edited by Simon Grabowsky, Berlin, Boston: De Gruyter, **2021**, 199-212. https://doi.org/10.1515/9783110660074-0085.
5. te Velde, G.; Bickelhaupt, F. M.; Baerends, E. J.; Fonesca Guerra, C.; van Gisbergen, S. J. A.; Snijders, J. G.; Ziegler, T., Chemistry with ADF. *Journal of Computational Chemistry* **2001,** *22*, 931-967.
6. Fonesca Guerra, C.; Snijders, J. G.; te Velde, G.; Baerends, E. J., Towards an order-N DFT method. *Theoretical Chemistry Accounts* **1998,** *99*, 391-403.
7. *ADF2022, SCM, Theoretical Chemistry, Vrije Universiteit, Amsterdam, The Netherlands,* http://www.scm.com.
